# Allogeneic dendritic cell (DC) vaccination as an “off the shelf” treatment to prevent or delay relapse in elderly acute myeloid leukemia patients: results of Phase I/IIa safety and feasibility study

**DOI:** 10.1186/2051-1426-1-S1-P205

**Published:** 2013-11-07

**Authors:** Tanja de Gruijl, Saskia Santegoeds, Sandra van Wetering, Satwinder Kaur Singh, Anthony Hall, Arjan A van de Loosdrecht, Ada Kruisbeek

**Affiliations:** 1 Dept of Heamatology, VU medical Centre, Amsterdam, the Netherlands; 2 Dept of Medical Oncology, VU medical Centre, Amsterdam, the Netherlands; 3 DCPrime, Leiden, the Netherlands

## 

A novel immunotherapy platform is represented by the DCOne cell line, which originates from a human myeloid leukemia cell line, endogenously expresses a range of leukemia associated antigens, including PRAME and WT-1, and can be differentiated into mature dendritic cells (DC). This cell line is being developed to replace patient-derived DC vaccines. The first indication in which mature DC derived from DCOne have been tested clinically is Acute Myeloid Leukemia (AML). A Phase I/IIa study enrolled 12 AML patients (age range 58-71) who were either in CR1/CR2 (n=5) or had smouldering disease (n=7). Patients had received all available standard care, were at high risk of relapse and ineligible for available post-remission therapies. A typical 3+3 design was used, starting with 4 bi-weekly intradermal DCOne DC vaccinations of 10E6 (n=3), 25E6 (n=3) or 50E6 cells(n=6). Patients were monitored for clinical and immunological responses for 126 days. Primary endpoints were safety and feasibility, and secondary endpoints were clinical and immunological responses. Treatment was well tolerated in all patients, with only injection site reactions (< grade 2). During the study 3 patients died: 2 from infections and 1 from leukemia. Clear evidence for induction of multi-functional immune responses was obtained, including increased post vaccination delayed type hypersensitivity reactions, increases in CD4+ and CD8+ T cell proliferative responses and/or seroconversion to DCOne DC and/or AML blasts in 6 out of 9 patients. Also, 3 out of 7 patients that were evaluable by IFNγ ELIPOTs showed vaccination-induced specific T cell responses to WT-1 and/or PRAME. Patients who survived more than 6 months post-vaccination showed remarkable survival (4 patients are still alive 27, 21, 12 and 9 months after study entry). One of these patients, with smouldering AML at entry, achieved CR2 after vaccination; one patient who was in CR2 at entry relapsed 8 months after vaccination, entered CR3 following a single low dose of 5-AZA and survived until 23 months. The two most recently enrolled patients recruited are still in remission at 12 and 9 months. We conclude that vaccination with DCOne derived DC is feasible in AML, and generates both cellular and humoral immune responses. The hypothesis that DCOne-derive DC induce immune responses against patient leukemia cells correlate with clinical benefit, will now be investigated in a multi-center randomized Phase II trial in AML patients in first remission.

